# Corticospinal Excitability Is Lower During Eccentric Than Concentric Cycling in Men

**DOI:** 10.3389/fphys.2022.854824

**Published:** 2022-03-16

**Authors:** Pierre Clos, Adrien Mater, Hippolyte Legrand, Gabriel Poirier, Yves Ballay, Alain Martin, Romuald Lepers

**Affiliations:** INSERM UMR 1093-CAPS, Université Bourgogne Franche-Comté, UFR des Sciences du Sport, Dijon, France

**Keywords:** motor-evoked potential, M-wave, silent period, knee extensor muscles, transcranial magnetic stimulation

## Abstract

How corticospinal excitability changes during eccentric locomotor exercise is unknown. In the present study, 13 volunteers performed 30-min strenuous concentric and eccentric cycling bouts at the same power output (60% concentric peak power output). Transcranial magnetic and electrical femoral nerve stimulations were applied at exercise onset (3rd min) and end (25th min). Motor-evoked potentials (MEPs) amplitude was measured for the rectus femoris (RF) and vastus lateralis (VL) muscles with surface electromyography (EMG) and expressed as a percentage of maximal M-wave amplitude (M_MAX_). EMG amplitude 100 ms prior to MEPs and the silent period duration were calculated. There was no change in any neural parameter during the exercises (all *P* > 0.24). VL and RF M_MAX_ were unaffected by exercise modality (all *P* > 0.38). VL MEP amplitude was greater (26 ± 11.4 vs. 15.2 ± 7.7% M_MAX_; *P* = 0.008) during concentric than eccentric cycling whereas RF MEP amplitude was not different (24.4 ± 10.8 vs. 17.2 ± 9.8% M_MAX_; *P* = 0.051). While VL EMG was higher during concentric than eccentric cycling (*P* = 0.03), RF EMG showed no significant difference (*P* = 0.07). Similar silent period durations were found (RF: 120 ± 30 ms; VL: 114 ± 27 ms; all *P* > 0.61), but the silent period/MEP ratio was higher during eccentric than concentric cycling for both muscles (all *P* < 0.02). In conclusion, corticospinal excitability to the knee extensors is lower and relative silent period longer during eccentric than concentric cycling, yet both remained unaltered with time.

## Introduction

An eccentric contraction is a forcible lengthening of the muscle-tendon unit while a concentric contraction consists in an active shortening. At the same force level, the former requires lower oxygen consumption (Abbott et al., 1952) and muscle activation (measured by surface electromyography—EMG; Bigland-Ritchie and Woods, 1976) than the latter. This appears to be due to the large contribution of passive structures, such as tendons or titin, to eccentric force production (Herzog, 2018). In addition, single-joint eccentric contractions have long been considered to require a unique neural control (Duchateau and Enoka, 2016). Notably, electroencephalography (Fang et al., 2001) or near-infrared spectroscopy (Borot et al., 2018) revealed that brain movement-related neural networks are more activated during eccentric than force-matched concentric contractions (Perrey, 2018). This greater brain activity is counterbalanced by inhibitions at the spinal levels, explaining the lower EMG level during eccentric than concentric contractions at the same torque level (Duchateau and Enoka, 2016).

Researchers have used transcranial magnetic stimulation (TMS) during single-joint eccentric contractions to test the excitability of the corticospinal tract—the primary descending neural pathway involved in voluntary movements (Lemon, 2008). They found corticospinal excitability (i.e., the amplitude of motor-evoked potentials—MEPs—normalized to the size of the maximal response evoked by electrical stimulation of the motor nerve—maximal M-wave, M_MAX_) to be lower during eccentric compared with concentric plantar flexions at the same force level (Duclay et al., 2014). This result is nuanced for the knee extensor muscles, with MEPs recorded on the vastus medialis (VM) and rectus femoris (RF) muscles being similar and smaller, respectively, during eccentric compared with concentric contractions at the same force level (Garnier et al., 2018). Furthermore, the duration of EMG interruption following MEP, termed silent period, is proportional to MEP amplitude and informs on inhibitory processes owing to both spinal and intra-cortical mechanisms (Yacyshyn et al., 2016; Škarabot et al., 2019). While the silent period is generally shorter during eccentric than concentric single-joint contractions (Duchateau and Enoka, 2016), the relative silent period (silent period duration/MEP amplitude ratio reflecting the balance between excitatory and inhibitory processes along the corticospinal pathway) is often greater during eccentric single-joint contractions (soleus muscle; Duclay et al., 2011)). Yet again, this result is not confirmed for the knee extensor muscles (Garnier et al., 2018).

To our knowledge, only one study assessed corticospinal excitability throughout a fatiguing eccentric single-joint exercise (i.e., during the dynamic contractions). Garnier et al. (2018) had participants complete a fatiguing exercise consisting of 10 series of 10 concentric or eccentric leg extensions at an intensity corresponding to 80% of their maximal isometric force. Applying TMS at the same knee flexion angle during the dynamic contractions, at the onset and end of the exercise, the authors did not observe any modulation of MEP size, EMG level 100 ms prior to MEPs or silent period duration for the VM and RF muscles. However, as argued by Sidhu et al. (2013), single-joint and locomotor exercises require separate scrutiny. Indeed, homeostasis is disturbed to a greater extent during locomotor exercises (elevated internal temperature or increased breathing), and central pattern generator may be involved (Forssberg et al., 1977).

Eccentric contractions can also be performed in the form of locomotor exercises, which are increasingly used in rehabilitation settings, thanks to their low energy cost. Particularly, eccentric cycling (i.e., pedaling backward resisting the torque of an engine) has been serving as an alternative to conventional cycling rehabilitation among cardiorespiratory-limited patients (Hoppeler, 2016). It has also been speculated that eccentric locomotor exercises could be a substitute to their concentric counterpart for neurorehabilitation (e.g., following a stroke; Clos et al., 2021). The rationale for this conjecture is as follows: while conventional locomotor exercises may trigger hemodynamic-related neuroplastic mechanisms (Knaepen et al., 2010), eccentric locomotor exercises, by the repeated intense activation of motor neural networks, may promote neuroplasticity as well. Despite its proven and potential benefits, the physiological responses to eccentric cycling remain partly unclear. Particularly, how a prolonged eccentric locomotor exercise, such as used in rehabilitation protocols challenges the neural system remains unknown (Clos et al., 2019).

Although changes in corticospinal excitability have not been assessed during a fatiguing eccentric locomotor exercise, authors successfully applied TMS at a constant crank angle during arm cycling (Zehr et al., 2007) or conventional lower-limb cycling (Sidhu et al., 2012b; Weavil et al., 2015). Using this method, Sidhu et al. (2012a) found the size of MEPs on the VL and RF muscles to remain stable from the beginning to the end of a 30-min fatiguing cycling bout (75% peak power output, reached with an increment of 30 W/3 min). Nonetheless, the authors proposed that the excitability of the corticospinal tract (MEP amplitude, normalized to M_MAX_ amplitude) does not drop throughout a fatiguing concentric cycling bout because of a rise in muscle activation (EMG amplitude, normalized to maximal EMG amplitude during a maximal voluntary isometric contraction). Indeed, considering EMG level at the angle of stimulation (during the pedaling cycles without TMS), the MEP amplitude/EMG amplitude ratio was reduced. In addition, another study of Weavil and colleagues (Weavil et al., 2016) supported this hypothesis by showing an increased MEP size with increasing EMG, in the absence of fatigue, but no increase in MEP for the same increase in EMG in the presence of fatigue. However, the same team (Sidhu et al., 2018) reported no change in VL MEP from before to immediately after a ∼9-min exhausting cycling bout (80% peak power output, reached with an increment of 25 W/min), assessed at the same EMG level. Since without EMG level rise, corticospinal excitability was expected to decrease due to fatigue, how muscle activation relates to corticospinal excitability in the presence of fatigue remains unclear.

As mentioned earlier, corticospinal excitability during a fatiguing eccentric cycling bout has not yet been described. The goal of the present study was thus to evaluate changes in corticospinal excitability and associated parameters (i.e., silent period duration, background EMG level) from the onset to the end of strenuous eccentric and concentric cycling bouts performed at the same power output. Here, we use the term “strenuous” to avoid confusion as the term “fatigue” is frequently used to indicate a decrease in maximal voluntary isometric force, which we did not measure in the current work. We hypothesized that MEP amplitude (% M_MAX_) would not be altered during either exercise, yet background EMG (% M_MAX_) would increase more during eccentric cycling, as recently found for the vastus lateralis muscle in an identical protocol (Clos et al., 2022).

## Materials and Methods

### Participants

Thirteen men (29 ± 8 years old, 1.78 ± 0.05 m, 68.2 ± 7.4 kg) participated in this study, which was conducted in accordance with the declaration of The World Medical Association [WMA] (n.d.). All participants gave a written informed consent. The study was approved by the French ethics committee (CPP EST I) and was registered on ClinicalTrials.gov (Identifier: NCT03280875).

### Study Design

**FIGURE 1:**
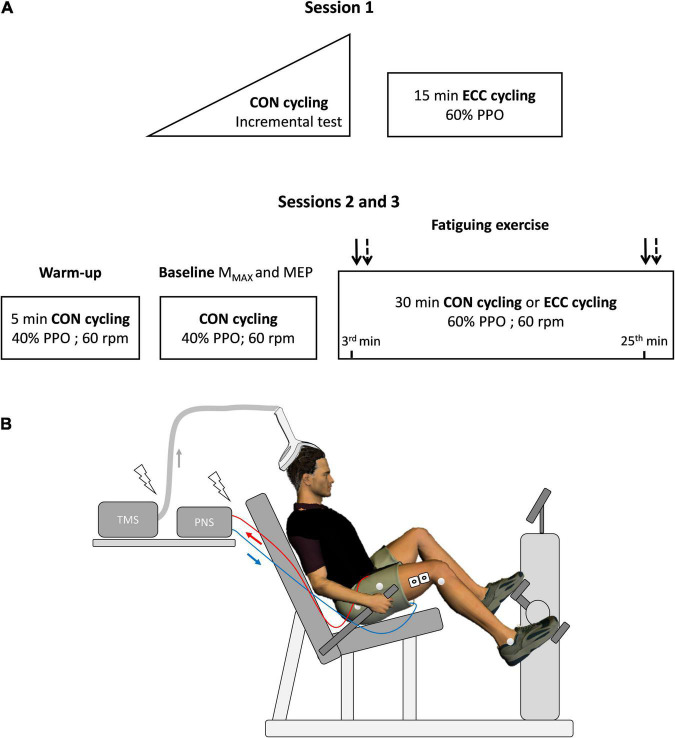
Overview of the experimental protocol. In **(A)**, a first session served to determine peak power output (PPO, W) during a concentric (CON) incremental cycling test before familiarizing participants with eccentric cycling (ECC). During the experimental sessions (2 and 3), maximal muscle action potential (M_MAX_) and motor-evoked potential (MEP) were obtained using electrical stimulation of the femoral nerve and transcranial magnetic stimulation (TMS), respectively. Baseline measurements lasted about 10 min. M_MAX_ (plain arrows) and MEP (dashed arrows), were also measured at the 3rd and 25th min of the strenuous exercises. Heart rate and effort perception were assessed at the same time points. rpm: rotations per minute. The experimental set-up is illustrated in **(B)**, with electromyographic electrodes on the vastus lateralis muscle (as well as the rectus femoris and vastus medialis, not represented in the figure) and reflective markers positioned directly on the skin on anatomical points chosen to calculate knee flexion angle. PNS: Peripheral nervous stimulation.

Figure 1 offers an overview of the experimental protocol. The participants came to the laboratory three times with 7–14 days of interval between each visit. On the first day, they completed an incremental concentric test to obtain their peak power output (PPO, W), followed by 15 min of eccentric cycling at 60% PPO (familiarization bout). Femoral nerve and transcranial stimulations were delivered while pedaling to further accustom the participants to the experimental procedures. In the next two sessions, completed in a random and counterbalanced order, stimulation intensities were set during concentric cycling at 40% PPO after a 5-min warm-up at the same intensity. Then, the participants pedaled for 30 min in either concentric or eccentric modalities at 60% PPO (described under “exercises”), an intensity and a duration found to induce similar knee extensor maximal voluntary isometric force loss in individuals familiar with eccentric cycling (Peñailillo et al., 2015; Clos et al., 2022). Motor nerve as well as TMS were delivered at a constant crank angle at the onset (after 3 min) and end of the exercise (after 25 min) —described under “electrical and magnetic stimulations.”

### Exercises

Two semi-recumbent cycle ergometers were used for concentric (Ergoline GmbH, Ergoselect 600, Bitz, Germany) and eccentric (Cyclus 2, Cyclus GmbH, Leipzig, Germany) cycling. Right knee flexion angle was measured using high-speed cameras (Vicon Motion Systems Ltd., England) at a 0°crank angle (upper vertical position) on each bicycle, and the seat distance and incline were adjusted so that there was fewer than 10° of difference in knee flexion angle between the two ergometers. The devices were set on isopower mode, adjusting the torque based on pedaling velocity. During the first visit, the incremental test started at 50 W with an increment of 1 W every 3 s and was performed at a free cadence. The two next sessions started with a 5-min warm-up and baseline measurements (10 min of total duration with about 6 min of pedaling and 4 min of rest between stimulation sets), both at 40% PPO—low intensity chosen to avoid the occurrence of fatigue prior to the 30-min exercises. Then participants performed the concentric and eccentric cycling exercises at 60% PPO. Pedaling cadence was visible to participants and they were instructed and frequently reminded to pedal at a rate of 60 ± 3 rpm. Indeed, as evidenced during concentric arm cycling, pedaling intensity and cadence both affected corticospinal excitability (Lockyer et al., 2018) and were thus critical to match between the two exercises.

### Electromyography and Kinematics

Bipolar wireless EMG units (Pico Cometa, Biometrics, France) were placed on Ag/AgCl electrodes (recording over 10 mm; center-to-center distance of 15 mm). The signal was obtained at a frequency of 1,800 Hz, amplified 200 times, stored for offline analysis and then band-pass filtered between 30 and 300 Hz. The skin of the participants was shaved and cleaned with alcohol swabs, and electrodes were positioned on the belly of the vastus lateralis (VL) and vastus medialis (VM) muscles and on the distal portion of the rectus femoris (RF) muscle, following the recommendations of Barbero et al. (2012). At the beginning of every session, electrodes were carefully replaced using anatomical landmarks.

The right knee flexion angle was instantaneously computed using 5 high-speed infrared cameras (300 Hz; Vicon Vero, Vicon Motion Systems Ltd., England) based on 4 reflective markers placed on the following anatomical locations: the great trochanter, the lateral tibial epicondyle, the thigh—10 cm distal from the great trochanter and the lateral malleoli.

### Heart Rate and Perception of Effort

Heart rate was obtained using a chest-belt monitor (model V800, Polar Electro Oy, Kempele, Finland) and written down by the experimenter at 3 and 25 min of exercise. At the same moments, participants reported their perception of effort (i.e., their difficulty to breathe and to drive their legs to pedal (Halperin and Emanuel, 2019), using Borg’s CR100 scale (Borg, 2007). Memory anchoring was performed with a rating of 100 on the CR100 scale, corresponding to the most intense effort participants had experienced running or pedaling.

### Electrical and Magnetic Stimulations

**FIGURE 2:**
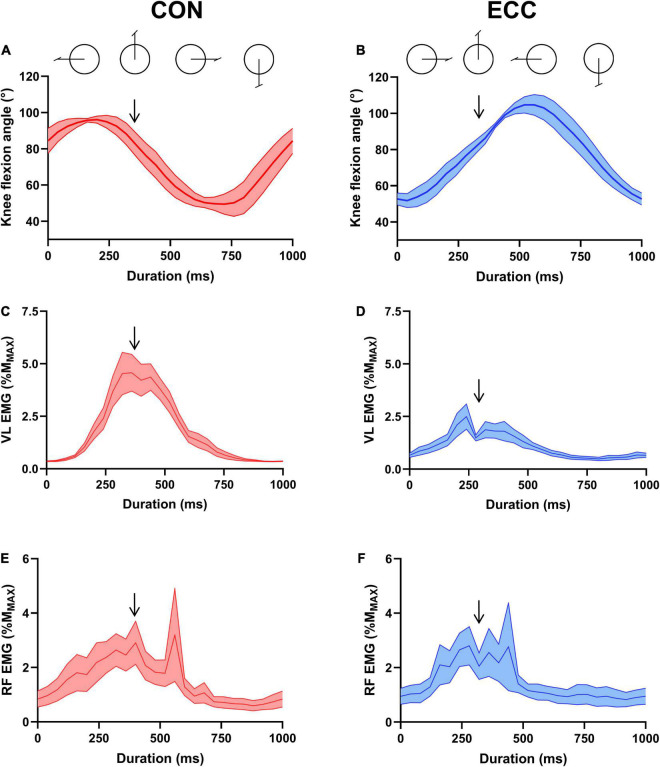
Knee flexion angle and neural drive across a pedaling cycle. **(A,B)** Illustrate knee flexion angle during concentric (CON) and eccentric (ECC) cycling, respectively. **(C)** (CON) and **(D)** (ECC) show vastus lateralis muscle EMG RMS and **(E)** (CON) and **(F)** (ECC) represent rectus femoris muscle EMG RMS. All data were obtained at the onset of the strenuous exercises, normalized to the amplitude of the maximal muscle compound (M_MAX_) and are represented as mean ± standard error. Downward arrows indicate the moment of peripheral and transcranial stimulations.

During pilot tests (*n* = 8), we used Nexus software 10.0 (Vicon Motion Systems Ltd., England) to apply femoral nerve stimulations at 4 knee flexion angles while participants were pedaling, corresponding to 4 crank angles at rest (0°, upper vertical position; 90° horizontal and forward; 180°, lower vertical; and 270°, horizontal and backward). We found that the 0°crank position was the only one allowing stimulations to fall within the EMG burst for all participants in both pedaling modalities. This crank position also allowed a very close knee flexion angle in both cycling modalities (concentric: 87.5 ± 4.7° vs. eccentric 88.5 ± 4.8°), which is critical given that knee flexion angle is known to influence corticospinal excitability (Doguet et al., 2017). Consequently, all stimulations during the cycling exercises were delivered at a crank angle of 0° (Figure 2). Electrical *s*timulations were administered using a constant-current stimulator (Digitimer DS7, Hertfordshire, United Kingdom). The device was connected to a circular cathode (3 cm in diameter) positioned upon the femoral nerve and to a rectangular anode (8 × 4 cm) in the gluteus fossae. TMS (Magstim, Whitland, Dyfed, United Kingdom) was applied to the motor cortex area contralateral to the right knee extensors, using a double-coned coil and a posterior-to-anterior current flow direction. The optimal coil position (“hotspot”) was determined by delivering 5 stimulations in different positions and observing which gave the highest average peak-to-peak MEP amplitude (Garnier et al., 2017).

Femoral nerve electrical stimulations intensity (0.2 ms, 400 V, 343.8 ± 80.1 mA) was increased until the EMG response of all muscles plateaued (M_MAX_) and then increased again by 20%. TMS intensity was set to obtain MEPs that were clearly distinguishable from background EMG and above 25% M_MAX_ for the VL muscle. Intensities were very close between the two sessions (50 ± 6.6 and 49.9 ± 6.3% maximal stimulator output). Four participants did not tolerate strong TMS and the stimulations were delivered at the highest bearable intensity. Sets of 4 femoral nerve stimulations and 15 TMS (from 5 to 10 s between pulses) were then delivered during leg cycling when the leg passed the 0°crank position to determine baseline muscle and corticospinal excitability (40% PPO—to make sure stimulations led to similar responses between the two sessions), as well as after 3 min and 25 min within each exercise condition (60% PPO).

### Data Analyses

Heart rate was expressed as a percentage of the peak value reached during the incremental test. Of the four peripheral nerve stimulations delivered at each time-point, the two giving the greatest peak-to-peak amplitude (Besomi et al., 2020) on the VL muscle were kept and averaged (M_MAX_). The EMG root mean square (RMS) was calculated 100 ms prior to each TMS stimulation artifact for all muscles. MEP amplitude and EMG RMS (*n* = 13) were both expressed as a percentage of M_MAX_ at the same time point in order to take muscle excitability into account. Thus, the EMG level (% M_MAX_) offered an estimation of neural drive (i.e., motoneuronal output; McManus et al., 2021). Silent period duration was measured manually from TMS stimulation artifact to EMG resumption in participants showing a clear EMG resumption (*n* = 10), using the criteria depicted by Damron et al. (2008). Among these participants, silent period durations with unclear limits were not measured. For MEP amplitude and silent period duration, data that were more than 2.5 standard deviations above or below the mean value were removed (8.9% of data). After this sorting, 13.3 ± 1.3 MEP amplitude and 12.1 ± 1.9 silent period duration values per stimulation set and per muscle (VL and RF) remained. MEP amplitude/EMG RMS (MEP/EMG) ratios and relative silent period (SP/MEP, ms/mV; Orth and Rothwell, 2004) were calculated based on the average within each individual.

### Statistical Analyses

Analyzes were carried out on Jamovi (version 1.6.23).^1^ Distribution normality was assessed with Shapiro-Wilk test. Baseline M_MAX_ and MEP were compared with Student’s *t*-tests, and two-way repeated measure ANOVAs (TIME—start, end—CONDITION—concentric, eccentric) were used for normal data collected during the exercises (heart rate, effort perception, M_MAX_, MEP, SP, SP/MEP). ANOVA *P*-values were adjusted for sphericity with Greenhouse-Geisser method. Tukey HSD *post-hoc* tests were applied in case of a significant main effect (*P* < 0.05). RF and VL background EMG, as well as MEP/EMG ratios, were treated with Friedman ANOVAs and followed-up by Durbin-Conover pairwise comparisons if significance was reached. *P*-values were corrected using Holm-Bonferroni method. Pearson correlation coefficient served to assess the repeatability of M_MAX_ (mV) and MEP (% M_MAX_) amplitudes between the baseline concentric bout of each session. Partial eta squared and Cohen’s dz (Cohen, 2013) provided an estimation of effect sizes for ANOVA main effects and follow-up tests, respectively (0.2 < small effect < 0.5 < medium effect < 0.8 < large effect).

## Results

All results are expressed as mean ± standard deviation in the text and the table. The results of the VM muscle were similar to those of the VL muscle and can be found in supplementary material, along with full ANOVA results at the following doi: 10.6084/m9.figshare.16639396.

### Baseline Parameters

The mean concentric peak power output was 293.5 ± 54.2 W (4.3 ± 0.7 W/kg), with a maximal heart rate of 181.6 ± 6.3 bpm and a perception of effort of 95.3 ± 6.5 a.u. Table 1 shows the mean M_MAX_ and MEP values for the VL and RF muscles during concentric cycling prior to each strenuous bout.

**TABLE 1:** Muscle and corticospinal excitability per session prior to the 30-min bouts.

	M_MAX_ (mV)		MEP (% M_MAX_)	
	VL	RF	VL	RF
Concentric session	1.29 ± 0.6	1.04 ± 0.4	31.8 ± 13.6	25 ± 14.3
Eccentric session	1.42 ± 0.7	1.06 ± 0.4	30.5 ± 15.1	25.4 ± 12.6
*P*-value	0.56	0.9	0.62	0.89
Pearson’s r	0.26	0.39	0.78	0.63

*Maximal muscle compound (M_MAX_) and motor-evoked potential (MEP) of the vastus lateralis (VL) and rectus femoris (RF) muscles during concentric cycling at 40% peak power output, prior to the strenuous exercises. Of note, the small wave amplitudes are due to the unusually low electrode gain of 200.*

### Exercise Description

Figure 2 shows knee flexion angle, VL and RF EMG level across a concentric and eccentric pedaling cycle at the onset of exercise.

Average heart rate was greater during concentric compared with eccentric cycling (82 ± 8.4 vs. 54.4 ± 8.7% maximal heart rate; *P* < 0.001; $\eta_{p}^{2}$ = 0.84) and increased with TIME (from 63.5 ± 14.5% to 73.8 ± 16.5% maximal heart rate; *P* < 0.001; $\eta_{p}^{2}$ = 0.83) but showed no interaction effect (*P* = 0.08; *$\eta_{p}^{2}$* = 0.12). Perception of effort showed an interaction effect (*P* = 0.021; $\eta_{p}^{2}$ = 0.83). It went from 31.3 ± 11.7 to 62.8 ± 20.1 a.u. (*P* < 0.001; *dz* = 1.36) during the concentric session and from 13.7 ± 8.2 to 28.7 ± 9.9 a.u. (*P* < 0.001; *dz* = 1.55) during the eccentric session.

### Changes in Muscle and Corticospinal Excitability During the Exercises

**FIGURE 3:**
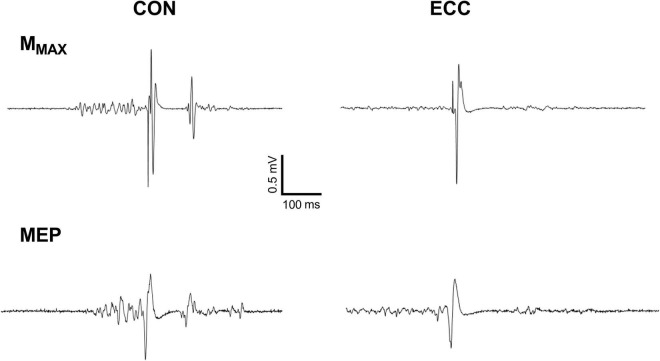
Raw traces of the response to femoral nerve and transcranial stimulations for the vastus lateralis muscle. The figure shows motor-evoked potential (MEP) and maximal M-waves (M_MAX_) during concentric (CON) and eccentric (ECC) cycling of a representative participant.

**FIGURE 4:**
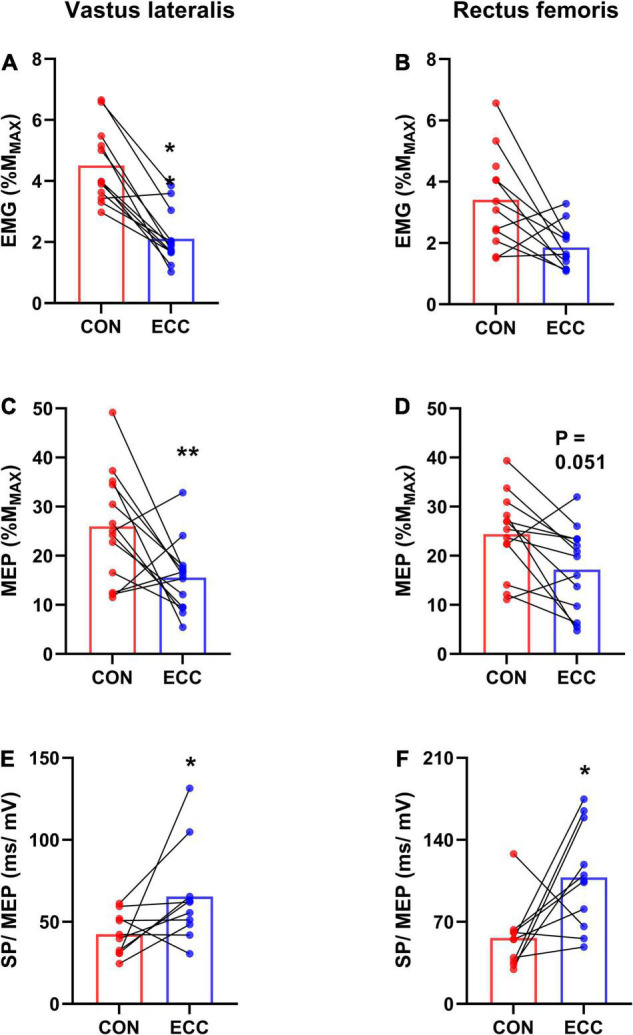
Corticospinal excitability and relative silent period. For each session (concentric—CON—or eccentric—ECC—cycling), this figure illustrates the average (mean of data from the onset and end of exercise) background EMG **(A,B)**, motor-evoked potential (MEP) amplitude **(C,D)** and the average ratio between silent period duration and MEP amplitude (SP/MEP) **(E,F)** for the vastus lateralis and rectus femoris muscles. *Indicates a difference between CON and ECC; one sign means *P* < 0.05; ** means *P* < 0.01.

Figure 3 displays raw M_MAX_ and MEP recorded on the VL muscle. Neither RF (0.96 ± 0.4 mV) nor VL (1.4 ± 0.6 mV) M_MAX_ amplitude was affected by TIME or CONDITION (all *P* > 0.38; all $\eta_{p}^{2}$ < 0.02). MEP amplitude (Figure 4) was greater during concentric compared with eccentric cycling for the VL (*P* = 0.008; $\eta_{p}^{2}$ = 0.27) but not the RF muscle (*P* = 0.051; $\eta_{p}^{2}$ = 0.15). Neither RF nor VL MEP amplitude changed with TIME (all *P* > 0.23; all $\eta_{p}^{2}$ < 0.06). RF EMG RMS prior to MEP was not significantly altered by exercise modality or from the onset to the end of exercise (Friedman’s *P* = 0.073). VL EMG RMS prior to MEP was greater during concentric than eccentric cycling (all *P* < 0.01; all *dz* > 1.3) but unaltered at the end of exercise (all *P* > 0.34; all *dz* < 0.2).

Neither the RF (Friedman’s *P* = 0.5) nor the VL (Friedman’s *P* = 0.24) MEP/EMG ratio was influenced by TIME or exercise modality (Table 2). RF (120 ± 30 ms) and VL (114 ± 27 ms) silent period duration did not differ between the onset and the end of exercise (all *P* > 0.38; all $\eta_{\text{p}}^{2}$ < 0.04) or between cycling modalities (all *P* > 0.61; all $\eta_{p}^{2}$ < 0.01). The amount of relative silent period (SP/MEP ratio; Figure 4) was greater for eccentric than concentric cycling for both muscles (all *P* < 0.033; all $\eta_{p}^{2}$ > 0.24) but remained stable across TIME (all *P* > 0.02; all $\eta_{p}^{2}$ < 0.09).

**TABLE 2:** Corticospinal excitability normalized to neural drive.

MEP/EMG ratio	Start of exercise		End of exercise	
Muscle	VL	RF	VL	RF
Concentric exercise	5.4 ± 1.9	9.1 ± 6.1	5.9 ± 3.6	7.4 ± 3.9
Eccentric exercise	8.5 ± 5.1	10.6 ± 5	6.2 ± 2.7	5 ± 1.8

*The table shows the MEP/EMG ratio (a.u.) of the vastus lateralis (VL) and rectus femoris (RF) muscles at the onset (3rd min) and end (25th min) of the strenuous concentric and eccentric cycling exercises.*

## Discussion

The aim of this study was to assess corticospinal excitability to the vastus lateralis (VL) and rectus femoris (RF) muscles during a 30-min strenuous concentric or eccentric cycling bout at the same power output. As expected, corticospinal excitability (i.e., MEP amplitude) remained stable during both exercises and for both muscles. Contrary to our hypothesis, the amount of neural drive (estimated by EMG level normalized to M_MAX_ amplitude) prior to MEPs remained unchanged as well. VL MEP amplitude was lower during eccentric than concentric cycling while RF MEP was not different. The MEP/EMG ratio was similar between exercise modalities and remained unchanged. Finally, while the duration of the silent period following MEPs was unaffected by exercise modality or from the onset to the end of the exercise, the SP/MEP ratio was higher during eccentric cycling, showing a longer inhibition relative to corticospinal excitability.

### Methodological Considerations

In this study, we did not measure the change in maximal voluntary isometric contraction force, which is the most commonly used marker of performance fatigability (Enoka and Duchateau, 2016), assuming it should decrease similarly after both exercises, such as previously reported by similar (Peñailillo et al., 2015) or identical protocols (Clos et al., 2022), as long as participants were familiar with eccentric cycling (Peñailillo et al., 2013). In fact, the aim of the study was not to probe neural modulations during the cycling exercises in relation with post-exercise isometric single-joint force decrease, but to assess the neural control of strenuous cycling exercises, *per se*. Since a stable power output induced a large increases in heart rate and effort perception during both exercise modalities, the exercises used in this study can be regarded as fatiguing (Enoka and Duchateau, 2016). However, as mentioned in the introduction, we use the term “strenuous” instead of “fatiguing” in the current article.

The data analysis procedure used in this study slightly differs from that used in previous investigations of corticospinal excitability during conventional leg cycling (Sidhu et al., 2012a; Weavil et al., 2016). Notably, although previous studies expressed EMG amplitude (i.e., muscle activation) as a percentage of the value during a maximal isometric contraction performed before the exercise (i.e., in the absence of fatigue), we opted for normalizing EMG amplitude to M_MAX_ amplitude at the same crank position. Despite different levels of amplitude cancelation between voluntary and evoked EMG signals (Besomi et al., 2020), taking muscle excitability changes into account allowed us to estimate neural drive (Duclay et al., 2014). Unlike previous studies on lower-limb cycling (Sidhu et al., 2012a), which considered the average EMG level in the absence of stimulations, we measured EMG level 100 ms prior to each transcranial stimulation artifact. While we consider both approaches to be valid (Lockyer et al., 2021), our choice of measuring the EMG before each stimulation was made because the size of each MEP should be influenced by the current neural drive, that is by EMG amplitude at the very moment of stimulation. Regardless, it must be noted that both analyses resulted in the same effects (or lack thereof) of exercise modality and TIME on background EMG amplitude and the MEP/EMG ratios.

### Corticospinal Excitability and Silent Period

Corticospinal excitability was higher during concentric than eccentric cycling for the VL muscle but not significantly different for the RF muscle (*P* = 0.051). Since for a given exercise modality, MEP amplitude is correlated with EMG amplitude (McNeil et al., 2011; Weavil et al., 2016), we calculated the MEP/EMG ratio. For the VL muscle, we found higher background EMG during concentric than eccentric cycling, but a similar MEP/EMG ratio. For the RF muscle, neither background EMG nor the MEP/EMG ratio were different between the two exercise modalities. These results suggest that corticospinal excitability (greater during concentric than eccentric cycling for the VL muscle and non-significantly different for the RF muscle) is related to neural drive level, regardless of exercise modality.

In single-joint settings, Garnier et al. (2018) found no difference in VM muscle activation (percentage of value during a maximal voluntary contraction) between contraction types, accompanied by no difference in corticospinal excitability. On the other hand, corticospinal excitability to the RF muscle and its activation were higher during concentric than eccentric leg extensions. Altogether, corticospinal excitability to the knee extensor muscles does not seem to depend upon contraction type or on whether the exercise mobilizes a single-joint or is locomotor, but to be determined by muscle activation level. The latter, however, varies according to exercise type and load (Alkner et al., 2000).

The facts that neither corticospinal excitability nor background EMG was affected at the end of the exercises partly contrast with the results of Sidhu et al. (2012a). The authors had participants perform 30 min of concentric cycling at an intensity similar to that used in the present study and found a decrease in the MEP/EMG ratio from the onset to the end of the exercise. This discrepancy may be related to the use of different cycling positions (i.e., upright vs. semi-recumbent) and/or pedaling cadences (80 rpm vs. 60 rpm). Together with findings from single-joint contractions (Garnier et al., 2018), our results indicate that corticospinal excitability to the knee extensor muscles remains stable during a strenuous dynamic exercise regardless of contraction type.

Similarly, the duration of the silent period, reflecting the duration of inhibitory processes along the corticospinal pathway (Škarabot et al., 2019), was stable and unaffected by exercise modality. While the relative silent period (Orth and Rothwell, 2004) was also unaltered at the end of the exercises, it lasted longer during eccentric than concentric cycling. In other words, a given excitability of the corticospinal pathway to the knee extensor muscles was accompanied by more pronounced inhibitory processes during eccentric compared with concentric cycling. This result diverges from single-joint knee extensions, where no difference was found between contraction types (Garnier et al., 2018), but is in accordance with most single-joint studies, showing a specific activation strategy during eccentric contractions (Duchateau and Enoka, 2016).

### Limitations

While normalizing EMG level to M_MAX_ amplitude allows to rule out the influence of muscle excitability on comparisons within cycling bouts and between modalities, it does not provide information upon the amount of muscle recruited relative to the voluntary maximum. The exercise intensity we chose was close to the one used by Peñailillo et al. (2017), who reported VL and RF muscle EMG to peak around 15% of the maximal voluntary EMG (obtained during an isometric contraction) during eccentric cycling. As for concentric cycling, the authors found VL muscle EMG to peak close to 35% and RF muscle EMG to peak at about 25% of maximal voluntary EMG.

In addition, MEP amplitude is known to augment with increasing contraction intensity (Sidhu et al., 2009) while silent period duration tends to decrease or remain stable (Škarabot et al., 2019). As a result, for the VL muscle the greater relative silent period during eccentric than concentric cycling may be due by the difference in muscle activation. Therefore, the use of a single power output level does not allow to conclude on a specific neural control of eccentric compared with concentric cycling.

### Perspectives

Thus far, the effects of exercise-induced fatigue on the neural control of an eccentric locomotor exercise remain elusive. Motoneuronal excitability, which seems to be lower during single-joint eccentric than concentric contractions at the same torque level (Grosprêtre et al., 2019), should also be assessed (by pyramidal tract stimulation) during fatiguing/strenuous eccentric cycling. Future research should also probe the mechanisms responsible for the more pronounced relative silent period during eccentric cycling. This may require the use of pyramidal tract stimulations within the silent period, such as implemented during conventional cycling by Sidhu et al. (2018).

## Conclusion

This study is the first to assess corticospinal excitability during a locomotor eccentric exercise. The results indicate lower corticospinal excitability and a longer silent period—relative to corticospinal excitability—during eccentric than concentric cycling at the same power output. However, these parameters seem to depend upon neural drive level, lower during eccentric cycling, rather than upon exercise modality in itself. None of these parameters were modulated from the onset to the end of the strenuous exercises. Future studies should investigate more precisely the mechanisms underlying the excitability of the corticospinal pathway at different levels (i.e., spinal vs. cortical) and whether these are modulated when performing a fatiguing exercise.

## Data Availability Statement

The raw data supporting the conclusions of this article will be made available by the authors, without undue reservation.

## Ethics Statement

The studies involving human participants were reviewed and approved by the Centre Hospitalier Universitaire Dijon. The patients/participants provided their written informed consent to participate in this study.

## Author Contributions

PC, RL, and AlM thought the experimental protocol and commented on the manuscript. PC, AdM, and HL collected the data. YB and GP fixed technical set-up issues. GP designed the initial script for data analysis. PC analyzed the data and drafted the manuscript. All authors approved the final version of the manuscript.

## Conflict of Interest

The authors declare that the research was conducted in the absence of any commercial or financial relationships that could be construed as a potential conflict of interest.

## Publisher’s Note

All claims expressed in this article are solely those of the authors and do not necessarily represent those of their affiliated organizations, or those of the publisher, the editors and the reviewers. Any product that may be evaluated in this article, or claim that may be made by its manufacturer, is not guaranteed or endorsed by the publisher.

## Footnotes

1
jamovi.org
